# Seasonality of *Vibrio parahaemolyticus* in the Eastern oyster, *Crassostrea virginica,* along the coast of Maine, USA

**DOI:** 10.1128/aem.00168-26

**Published:** 2026-07-31

**Authors:** Aidan Lurgio, Everett Rzeszowski, Amy Webb, Tyler Spillane, Stephen Jones, Sue Ishaq, Camille H. Ross, James Becker, Kohl Kanwit, Damian C. Brady

**Affiliations:** 1 Maine Department of Marine Resources, Bureau of Public Health and Aquaculture, Boothbay, Maine, USA; 2 School of Marine Sciences, University of Maine, Darling Marine Center 115973, Walpole, Maine, USA; 3 Department of Natural Resources and the Environment, University of New Hampshire 3067, Durham, New Hampshire, USA; 4 School of Food and Agriculture, University of Maine 115975, Orono, Maine, USA; Anses, Maisons-Alfort Laboratory for Food Safety, Maisons-Alfort, France

**Keywords:** *Vibrio parahaemolyticus*, Gulf of Maine, aquaculture, polymerase chain reaction, pathogenic, bacteria, generalized additive models

## Abstract

As the Gulf of Maine warms, characterizing seasonal baseline levels of the human pathogen *Vibrio parahaemolyticus* (*Vp*) in Eastern oysters (*Crassostrea virginica*) is crucial, along with synoptic environmental data, to develop predictive capacity and risk management strategies. In Maine, USA, from May to November 2023, oyster samples were collected from Scarborough River, Maquoit Bay, and the Damariscotta River. *Vp* was quantified using the U.S. Food and Drug Administration-approved three-tube most-probable-number (MPN) real-time PCR method, detecting the presence of total *Vp* (i.e., *tlh* gene) and potentially pathogenic *Vp* (i.e., *tdh* and/or *trh* genes). *tlh* was detected at all sites once water temperatures reached 16°C, with densities ranging from <3 to >1,100 MPN g^−1^. Generalized additive models revealed significant positive relationships between both potential pathogenic markers (*tdh* and *trh*) and sea surface temperature. *tdh* in the Damariscotta River, the largest oyster-growing area in northern New England, was the most responsive to water temperature, and *trh* in Maquoit Bay was the most responsive to salinity. Statewide *Vp* regulations for harvesting and handling oysters were implemented in Maine in 2024, and these baseline data already demonstrate the importance of maintaining cold chain storage, as potentially pathogenic strains were detected at all three sites. Given the growing number of *Vp*-associated illnesses and the clear response of *Vp* to warming conditions in the region, these baseline data provide timely insights for harvesters and inform policies to safeguard public health.

**IMPORTANCE:**

The Gulf of Maine is both a hotspot for expanding aquaculture production and one of the fastest warming marine regions in the world. Rising numbers of vibriosis illnesses in warmer months associated with consumption of raw oysters, including Maine’s first outbreak in 2023, highlight critical gaps in baseline data on the human pathogen *Vibrio parahaemolyticus* (*Vp*) (State of Maine, Department of Marine Resources, *Vibrio parahaemolyticus* control plan, chapter 115.02.a, 2016). The state of Maine requires up-to-date baseline information on *Vp* in productive growing areas to respond effectively to future illnesses and potential growing area closures. Our results support the need for a statewide *Vp* control plan that addresses harvest and post-harvest practices, particularly minimizing temperature abuse of live oysters. As climate change continues to warm coastal waters and alter estuarine conditions, systematic data on *Vp* abundance and seasonality are increasingly essential for managing the public health dimensions of shellfish aquaculture in Maine and similar high-latitude regions.

## INTRODUCTION

Shellfish aquaculture is rapidly expanding in the Gulf of Maine (GoM) (1). The extensive coastline provides ample opportunities to match the right aquacultured species to its optimal environmental conditions (2, 3). Aquaculture jobs in Maine more than doubled from 2016 to 2024. This growth was driven by shellfish farming, which is 18.5 times more concentrated in Maine than the national average (1). An increase in Limited Purpose Aquaculture sites from 85 to 769 from 2010 to 2024 and the number of aquaculture lease applications increasing from 18 in 2016 to 105 in 2022 provides further evidence of an industry in expansion (4, 5). Increasing shellfish aquaculture is a driving force for the economy—specifically due to the expanding interest in fresh Maine oysters. The Eastern oyster (*Crassostrea virginica*) is a dominant commercial species in the United States and is found along the Atlantic and Gulf coasts. From 2011 to 2021, landings for the Eastern oyster increased more than 10-fold from $1.2 million USD to just under $14 million USD (6, 7). However, oysters, like all molluscan bivalves, are filter feeders that can bioaccumulate microorganisms found in the water column. As such, consuming raw oysters comes with potential public health risks, as they filter bacteria such as *Vibrio parahaemolyticus*.

**Fig 1:**
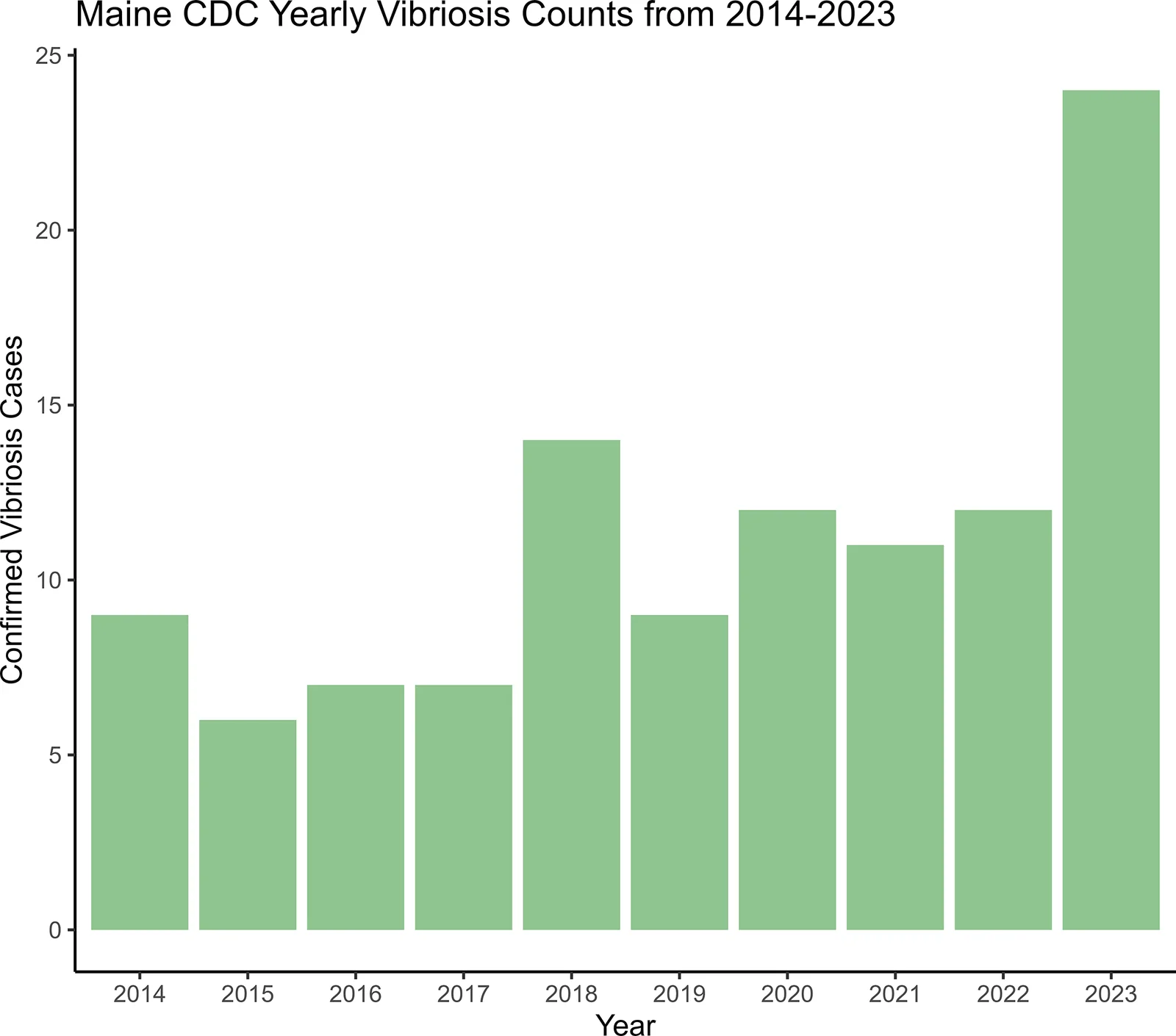
Vibriosis illnesses confirmed and reported to the Maine Center for Disease Control from 2014 to 2023. No reports of vibriosis illness are available before 2014.

*Vibrio parahaemolyticus* (hereafter referred to as *Vp*) is a halophilic, gram-negative bacterium ubiquitous in the marine environment and found within the water column–making it one such species of public health concern (8). Oysters concentrate *Vp* in their tissues and can accumulate bacteria at levels 100-fold higher than those in the surrounding water (9). Some strains of *Vp* are pathogenic to humans, causing gastroenteritis, commonly referred to as vibriosis, which results in nausea, diarrhea, and vomiting. *Vp* can also enter the body through a cut on the skin, causing wound infections (10). In rare cases, *Vp* can cause septicemia. First discovered in 1950 in Japan, with widespread distribution in this area by the 1960s (11, 12), *Vp* was detected in the United States during the late 1960s and in New England in 1970 (13) and is now the most frequent cause of vibriosis (14). Centers for Disease Control and Prevention (CDC) estimates that there are 45,000 *Vp* cases per year and that only 1 in 20 cases are reported (15). To estimate the annual number of episodes of foodborne illnesses in the United States, Scallen et al. (16) made the largest model adjustments for *Vibrio* species for underdiagnosis. Globally, *Vp* is the leading seafood infection with an estimated half-million cases in 2020 (17). In this warming marine environment, we see a greater number of human infections from *Vibrio* species (18). For example, Maine vibriosis case data from the ME CDC show the rate of illnesses increasing since 2014—doubling from 2022 to 2023 (Fig. 1). This recent year (2023) of increased *Vibrio*-related illnesses included Maine’s first *Vibrio fluvialis* outbreak from Maine-grown oysters and a corresponding growing area closure, which increased awareness and concern regarding this pathogen in Maine. *Vp* illnesses impact the aquaculture industry and nationally burden the healthcare system, costing an estimated $20 million per year (19). Increasing illness rates have spurred awareness throughout the industry and changes in state regulations.

Although found globally, the distribution of *Vibrio* species is shaped by environmental parameters, such as sea surface temperature (SST), salinity, pH, dissolved organic carbon, and seasonal blooms of zooplankton (20–22). *Vibrio*-related illnesses are commonly correlated with water temperatures of 15°C or above (23). *Vp* is found in waters with a salinity range of 10–34 psu but thrives in intermediate salinities between 20 and 30 psu (23, 24). Typically, salinity determines optimum environments, and temperature determines the abundance in those environments (25). Optimum water temperature and salinity levels lead to rapid population growth (17). *Vp* has one of the fastest growth rates of all estuarine bacteria; replication increases with water temperature, and the population is able to double itself every hour at 32°C (26). The Eastern oyster’s filtration rate is a strong function of temperature (8°C–30°C) (2), just as *Vibrio* density is a strong function of temperature (15°C–44°C) (27). With warming water temperatures, *Vp* increases, and oysters bioaccumulate this potential pathogen.

Climate change has direct impacts on human health, with changes in temperature and precipitation enhancing the spread of disease (28). Rising SST and changing salinities are the two most prominent abiotic effects of climate change in coastal areas, and *Vp* distribution is changing due to the presence of warmer and more saline coastal environments (25, 29). Trends resulting in the warming of 70% of the world’s oceans are expanding the optimal environmental range for *Vp* (17, 30). Harvell et al. (31) estimated that an average 1.5°C increase in seawater temperature would significantly extend the seasonal and geographic range of *Vp* abundance. Climate modeling suggests that pathogens like *Vp* will continue to be a threat to public health as waters warm, currents change, and precipitation increases (18). *Vp* is becoming more abundant in higher latitudes where it was historically less of a concern. In the last 3 years, the GoM has experienced record-breaking warming in response to anthropogenic climate change; the warmest years on record are 2021 and 2022, and 2023 is the fifth warmest year in recorded history (32). Recent rapid warming in the GoM due to a well-documented oceanographic regime shift in 2010 (33) is correlated with increasing vibriosis cases in the GoM (34), and Hartwick et al. (35) confirmed that SST was the dominant environmental factor associated with an increasing trend of *Vp* in Great Bay estuary, New Hampshire, USA oysters. It is clear that temperature conditions in the GoM have caused increased optimal conditions for oyster growth (6) while potentially simultaneously increasing the amount of time optimal for *Vp*. Enclosed bodies of water, such as estuaries, are warming even faster than the oceans (36). These unique coastal environments found throughout Maine are hotspots for oyster lease site selection (2). Warming estuaries mixed with changes in precipitation may result in increased suitable areas for *Vp* growth (25). Furthermore, the seasonality of vibriosis illnesses is associated with increased *Vibrio* abundance at harvest during the warmer months (14).

A reliable genetic marker found in nearly all strains and used for total detection of *Vp* is the thermolabile hemolysin gene (*tlh*) encoding phospholipase A2 (8, 37). *Vp* strains analyzed from clinical cases of human gastroenteritis often contain primary virulence factors, such as the pore-forming toxin, thermostable direct hemolysin (encoded by the *tdh* gene), *tdh*-related hemolysin (encoded by the *trh* gene), or both (23). *tdh*+ and *trh*+ strains are considered indicators of potentially pathogenic strains. *tdh* is an amyloid toxin that disrupts lipid microdomains, affecting cytotoxicity in gastrointestinal cells, which alter the ion flux in the intestine, proving to be the mechanism behind diarrhea associated with gastroenteritis; *trh* acts similarly by altering ion flux (38–40). Roughly 27% of all clinical isolates lack the *tdh* and *trh* genes, suggesting there are other important virulence factors (37, 41). Vibriosis cases in the Northeast United States, including outbreaks in New York, Connecticut, and Massachusetts, have coincided with warming SST and an invasion of Pacific native strains such as ST36, ST43, and ST636 into Atlantic coastal waters (42, 43). Accurate identification of rare pathogenic strains within populations of mostly non-pathogenic *Vp* has been an ongoing challenge (44). Researchers have described extra steps that include genotyping to identify strains and further development of assays that directly identify strains such as ST36 (44–48). *Vibrio* species are known to undergo recombination and utilize horizontal gene transfer, allowing for the transfer of genetic material between strains and species; this ability allows the bacteria to adapt to niches promoting speciation, which has encouraged the emergence of pandemic clones (37, 44, 49, 50). Through the acquisition of distinct *Vibrio* pathogenicity islands, Xu et al. (43) illustrated that some native strains can acquire foreign DNA and become human pathogens documented in the Northeast United States. While understanding what causes strains of *Vp* to be pathogenic is an ongoing area of research, associating *tdh* and *trh* strains with pathogenicity in humans remains a relatively consistent methodology (37, 51–56).

Human exposure to this pathogen cannot be completely eliminated, but if regulations related to shellfish handling and harvest practices are in place when environmental conditions encourage growth, the incidence of illness can be greatly reduced (53). To decrease the number of *Vibrio*-related illnesses in the United States, state agencies have implemented *Vp* control plans that vary by state. Although other pathogenic *Vibrio* species can be found, control plans specific to *Vp* can still be effective for minimizing all illnesses. The National Shellfish Sanitation Program (NSSP) provides guidelines regarding shellfish harvesting and handling practices, which aim to minimize bacterial growth in harvested oysters (57, 58). The U.S. Food and Drug Administration (FDA) level of concern for *Vp* in shellfish is ≥10,000 bacteria g^−1^ (59). The probability of infection even after consumption of this concentrated amount of bacteria is still considered relatively low (52, 58).

For the state of Maine, a *Vp* control plan has been in effect annually from 1 June to 5 October since 2016. Control plans aim to limit time from harvest to initial refrigeration since *Vp* can continue to grow and multiply in oysters until they are adequately chilled below 10°C, where growth is inhibited (51, 60). In 2023, there were six growing areas throughout the state of Maine designated as *Vp* control areas. An outbreak, defined as two or more *Vp* cases from one implicated area, will cause a growing area closure (58) and lead to economic losses for the shellfish industry during the busy summer months (34). Regulatory guidance recognizes that the presence of *tdh* and *trh* may cause an increased risk to public health (14). Using an approved NSSP method, an FDA-approved lab can test oysters from an implemented area. However, without baseline data (expected abundance from previous environmental monitoring), both pathogenic genes (*tdh* and *trh*) in these samples must not exceed 10 MPN g^−1^ (58). The lack of baseline data available for *Vp* in Maine fails to prepare the state for future closures. *Vp* abundance and the environmental conditions associated with this human pathogen impede the understanding of *Vibrio*-related illness and its link to ocean warming.

To characterize the baseline abundance and environmental drivers of total and potentially pathogenic *Vp* along the GoM, oysters and environmental data were collected from three distinct and productive growing areas for 7 months. The objectives were to (i) determine the abundance of the *tlh*, *tdh*, and *trh* genes found *in Vp* within Eastern oysters from three representative growing areas (Scarborough River, Maquoit Bay [MB], and Damariscotta River) to begin establishing the baseline levels of total and potentially pathogenic *Vp*, (ii) evaluate the relationships between *Vp* abundance and environmental parameters (i.e., temperature and salinity) between study sites, and (iii) use generalized additive models (GAMs) to assess the potential influence of environmental variables on the seasonal patterns of *Vp* abundance.

## RESULTS

**Fig 2:**
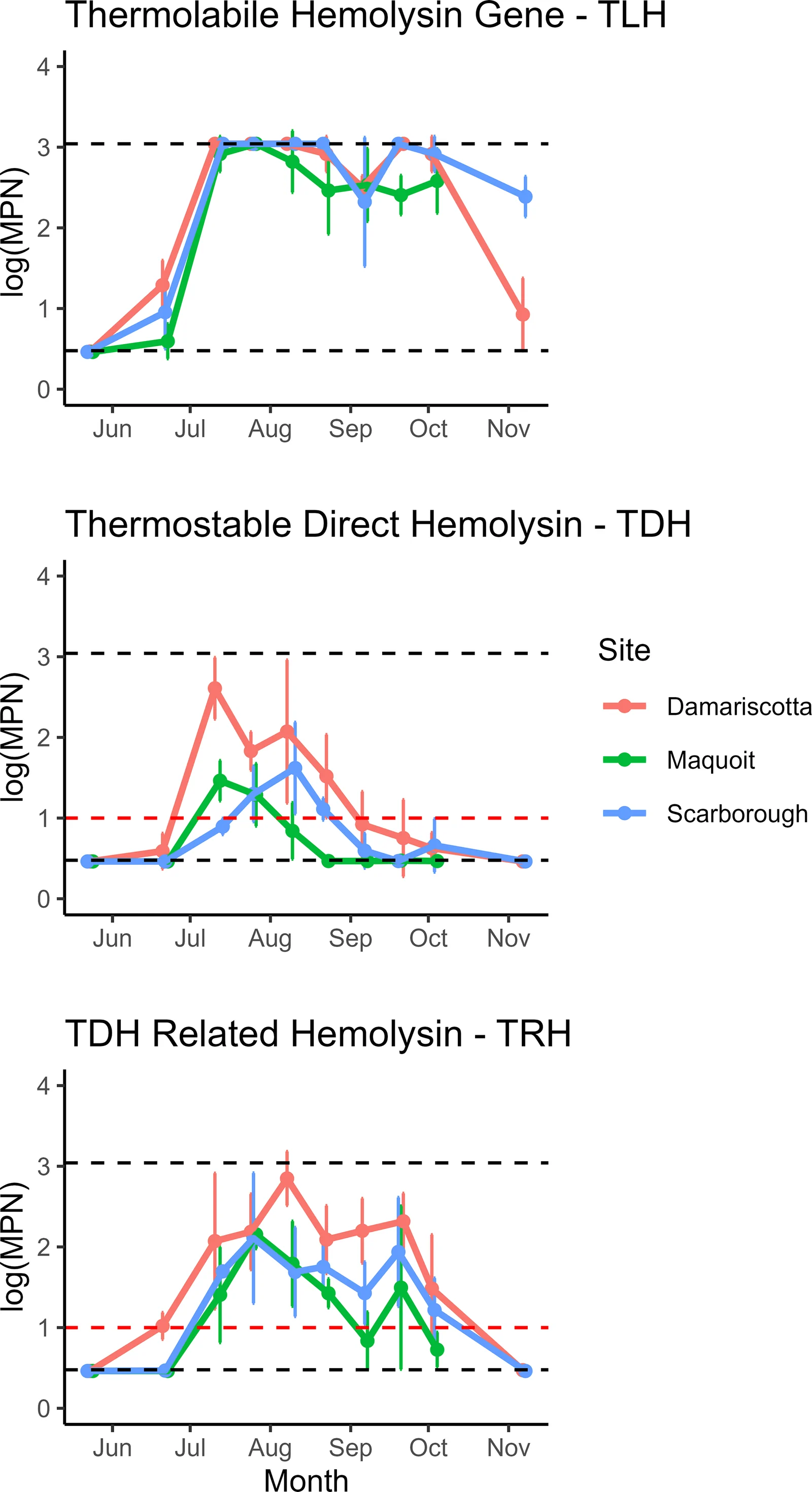
Abundance of *Vibrio parahaemolyticus* in log MPN g^−1^ in oysters from Scarborough River estuary, Maquoit Bay, and Damariscotta River estuary in Maine. Dots are representative of average abundance across triplicate samples, with error bars indicating the standard deviation. The dotted black lines show the minimum and maximum detection limits, and the dotted red line shows the FDA limit of *trh* and *tdh* in shellfish to reopen after an illness closure.

Analysis of 87 oyster samples showed *Vp* abundance ranging from <3 to >1,100 MPN g^−1^ (Fig. 2). The *tlh* gene, found in all strains of *Vp* and representing total abundance, was detected in 87% (76 out of 87) of all oyster samples (Table 1), exceeded the maximum detection limit (>1,100 MPN g^−1^) in 44% (38 out of 87) of the oyster samples, and was the most abundant gene with the highest mean PCR efficiency of 1.74 (perfect efficiency = 2.0). This dilution scheme only provided detection up to 1,100 MPN g^−1^, which was exceeded in 44% of the samples during peak abundance months; increased dilutions are necessary for accurate quantification in the future.

**TABLE 1:** Summary of results for the presence of *tlh*, *tdh*, and *trh* in all oyster samples

Site	No. of samples	Samples positive for *tlh*	Samples positive for *tdh*	Samples positive for *trh*
Damariscotta	30	27	22	26
Maquoit	27	23	14	20
Scarborough	30	26	17	22
Total	87	76	53	68

The *tdh* and *trh* genes represent two potentially pathogenic markers; *tdh* was less abundant throughout all three sites, and *trh* was the most abundant. The *tdh* probe had the lowest mean PCR efficiency, ranging between 1.41 and 1.59 (perfect efficiency = 2.0), exceeded the maximum detection limit in 1.15% (1 out of 87) of the samples, and was detected in 61% (53 out of 87). In contrast, the *trh* gene was detected in 78% (68 out of 87) of the samples (Table 1), exceeded the maximum detection limit in 4.6% (4 out of 87) of samples, and resulted in a mean PCR efficiency of 1.713 across all PCR runs. The *tdh* probe showed unusually low PCR efficiency, potentially affecting accurate abundance data. The *tdh* samples with consistently low efficiency are still retained to provide temporal coverage. The difference in PCR efficiency between genes likely means that *tdh* detection is underestimated within these sample results, and gene comparison should be interpreted cautiously; even so, we observed seasonal fluctuations that corresponded with changes in our environmental variables. The dilution scheme used was not accurate for potentially pathogenic targets during peak abundance months, as five samples exceeded detection, leading to unknown and crucial baseline data.

**Fig 3:**
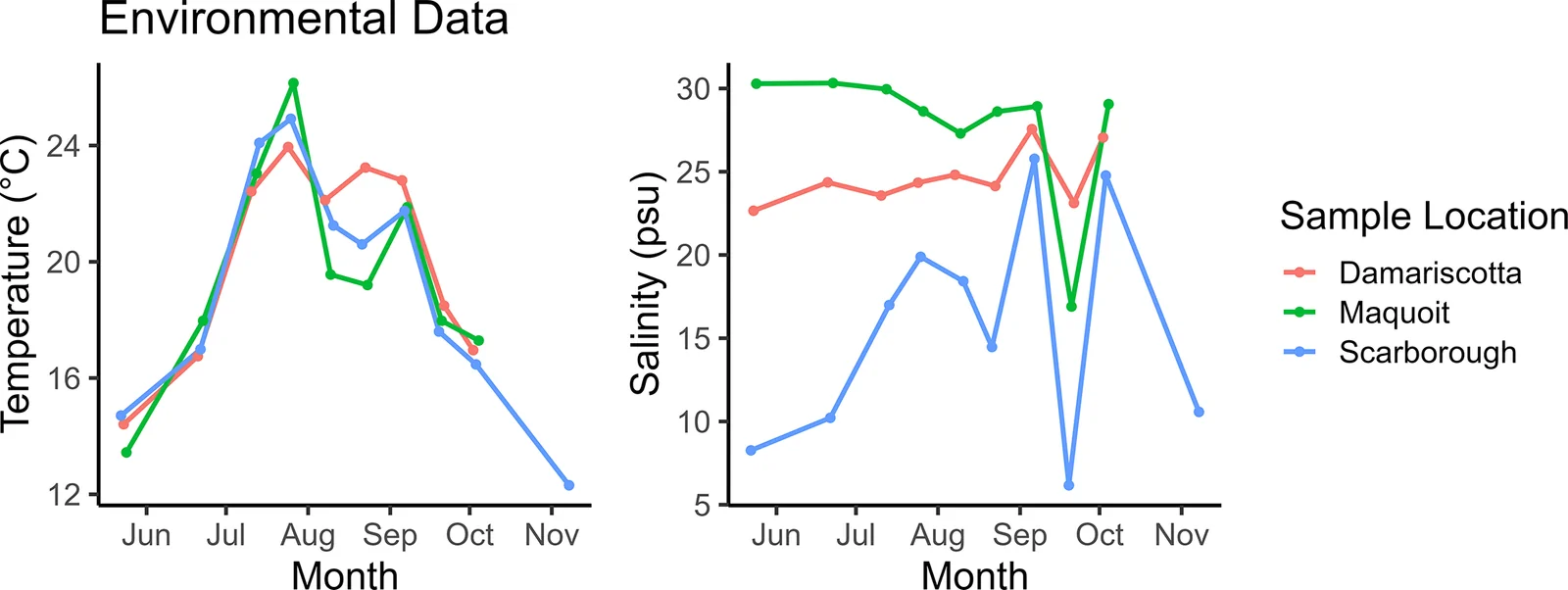
Temperature and salinity data from the three sites measured at the time of sample collection from a calibrated YSI meter.

Salinities varied between sites, and seasonal trends in water temperature were the primary driver of *Vp* abundance. Environmental conditions at the time of each sample showed salinities ranging between 6 and 30 across all sites, while water temperatures ranged between 12°C and 26°C (Fig. 3). *Vp* thrives in salinity between 20 and 30 psu and water temperatures between 15°C and 44°C (18, 24, 52). The warmest water temperatures during sampling were observed during the second sample in July, when MB reached 26°C and when the maximum *trh* abundance at this site was observed (Fig. 2 and 3). Each location showed a decrease in salinity levels on the week of 19 September 2023 compared to the samples from 2 weeks before and 2 weeks after, corresponding to a rainfall event (61). Scarborough River estuary (SRE) and MB decreased in salinity levels more so than Damariscotta River estuary (DRE) due to increased river discharge in these two sites; salinity levels in the SRE dropped from 25 to 6 psu, and levels in MB dropped from 30 to 7 psu, which correlated with an increase in *tlh* and *trh* abundance. Hourly temperature means from May to November across sites were significantly different (chi-squared = 1,205.8, df = 2, *P*-value < 0.05), and salinity values were also significantly different (chi-squared = 3,528.3, df = 2, *P*-value < 0.05).

### Scarborough River estuary

Throughout the entire sampling season, the SRE ranged in temperature from 8.6°C to 25.8°C with a mean of 17.5°C. Salinity at the time of sampling ranged from 6.2 to 24.8 psu. Compared to the other sites, SRE maintained the lowest average salinity across sampling events. The total *tlh* gene was detected in 86.6% (26 out of 30) of samples and was >1,100 MPN g^−1^ in 60% (18 out of 30) of samples. *tdh* was detected in 57% (17 out of 30) of samples and never exceeded the detection limit. *trh* was detected in 73% (22 out of 30) of samples and was >1,100 MPN g^−1^ in 3% (1 out of 30) of the samples.

A seasonal change in all three genes was observed from May to November. *tlh* and *trh* began to increase once water temperatures rose above 15°C. However, all three genes increased once water temperatures rose above 20°C. Conversely, there was a dramatic decrease once temperatures dropped below 15°C again by November. Detection of *trh* began in June with one sample detecting 3 MPN g^−1^. Detection of *tdh* began in the first sample of July. The most abundant *trh* detection occurred during the second sample in July when one sample was above the detection limit (>1,100 MPN g^−1^). At the time of this sample, salinity reached 19.9 psu, and the warmest site-specific sampling temperature of 24.9°C was recorded. The most abundant sample of *tdh* was detected in the first sample of August. By the final sample, in November, both *tdh* and *trh* were undetectable.

Overall, analysis of potentially pathogenic strains *tdh* and *trh* allowed for an understanding of the seasonality of *Vp*. In one sample, *trh* was detected above the maximum; besides this second sample in July, we were able to observe the full range of *Vp* abundance in this area.

### Maquoit Bay

Throughout the sampling season, MB ranged in temperature from 11.5°C to 27.2°C with a mean of 19.4°C. Salinity at the time of sampling ranged from 16.9 to 30.3 psu. This low-end salinity value of 16.9 psu was only observed on 20 September 2023, and the next lowest level was 27.3. Compared to the other sites, MB maintained a higher average salinity across sampling events. An oyster sample from November could not be collected from this site. *tlh* gene was detected in 85.1% (23 out of 27) of samples and was >1,100 MPN g^−1^ in 15% of samples (4 out of 27). *tdh* was detected in 52% (14 out of 27) of samples and never exceeded the detection limit. *trh* gene was detected in 74% (20 out of 27) of samples and never exceeded the detection limit.

Seasonal dynamics at MB generally paralleled SRE, with some notable exceptions. There was an early peak in the detection of *tdh* at MB compared to the SRE, where all three genes began to decline earlier. Additionally, during sampling, water temperature peaked at the MB site, but it was also the first of the estuarine waters to drop below 20°C in August. Detection of *tlh* started in June, and *tdh* and *trh* were detected starting in the first sample in July. Detection of all three target genes peaked in abundance at this site in late July when salinity reached 28.6 psu and the warmest water temperature of 26.2°C at the time of the sampling was recorded. By the final sample in October, *tdh* and *trh* had decreased for the season, both being detected below the FDA reopening limit of 10 MPN g^−1^. *tlh* remained abundant and had not yet decreased by October for the season.

A lack of a sample in November impeded our ability to understand when *Vp* was no longer detected in this area. With only 15% of samples reaching the maximum detection for *tlh*, we were almost able to observe the abundance of total *Vp* in this area and know that maximum detection was in July. Although, compared to the other two sites, no sample for either *tdh* or *trh* reached maximum capacity, we were able to evaluate the full extent of potential pathogenic *Vp* abundance in this area.

### Damariscotta River estuary

Throughout the entire sampling season, the DRE ranged in temperature from 11.9°C to 27.1°C with a mean of 20.2°C, showing that on average, DRE was 0.8°C–2.2°C warmer than MB and SRE. Salinity at the time of sampling ranged from 22.6 to 27.5 psu. Environmental data for November were not available for this site. Total *tlh* was detected in 90% of samples (27 out of 30) and exceeded the detection limit (>1,100 MPN g^−1^) in 53% (16 out of 30) of samples. *tdh* was detected in 73% of samples (22 out of 30) and exceeded the detection limit (>1,100 MPN g^−1^) in 3% (1 out of 30) of samples. *trh* was detected in 87% of samples (26 out of 30) and exceeded the detection limit in 10% (3 out of 30) of the samples.

Similar seasonal cues were observed in the DRE as in the other two sites, but *Vp* was significantly more abundant. The *tlh* gene was detected in one sample in May at 3 MPN g^−1^. In June, both *tdh* and *trh* genes were detected, and by July, *tdh* and *trh* genes were reaching or exceeding 1,100 MPN g^−1^. *Vp* abundance peaked at this site in early August when salinity reached 24.8 psu and the temperature was 22.1°C. *tdh* and *trh* peaked at different times; *tdh* peaked in early July, while *trh* peaked in early August. Although the DRE did not reach as warm a temperature at the time of the sampling as the other two sites, it maintained a warmer sampling temperature in August, September, and October and had a warmer mean SST determined by continuous monitoring with the Hobo U24 data logger. By November, *tdh* and *trh* genes were at or below the minimum detection limit of 3 MPN g^−1^ (Fig. 2).

We observed the most abundant detection of potentially pathogenic strains in DRE compared to the other two areas. Due to the limitations of comparisons in *tlh*, we cannot confirm whether the DRE also has increased total *Vp* compared to SRE. More dilutions are necessary to fully evaluate this growing area.

**Fig 4:**
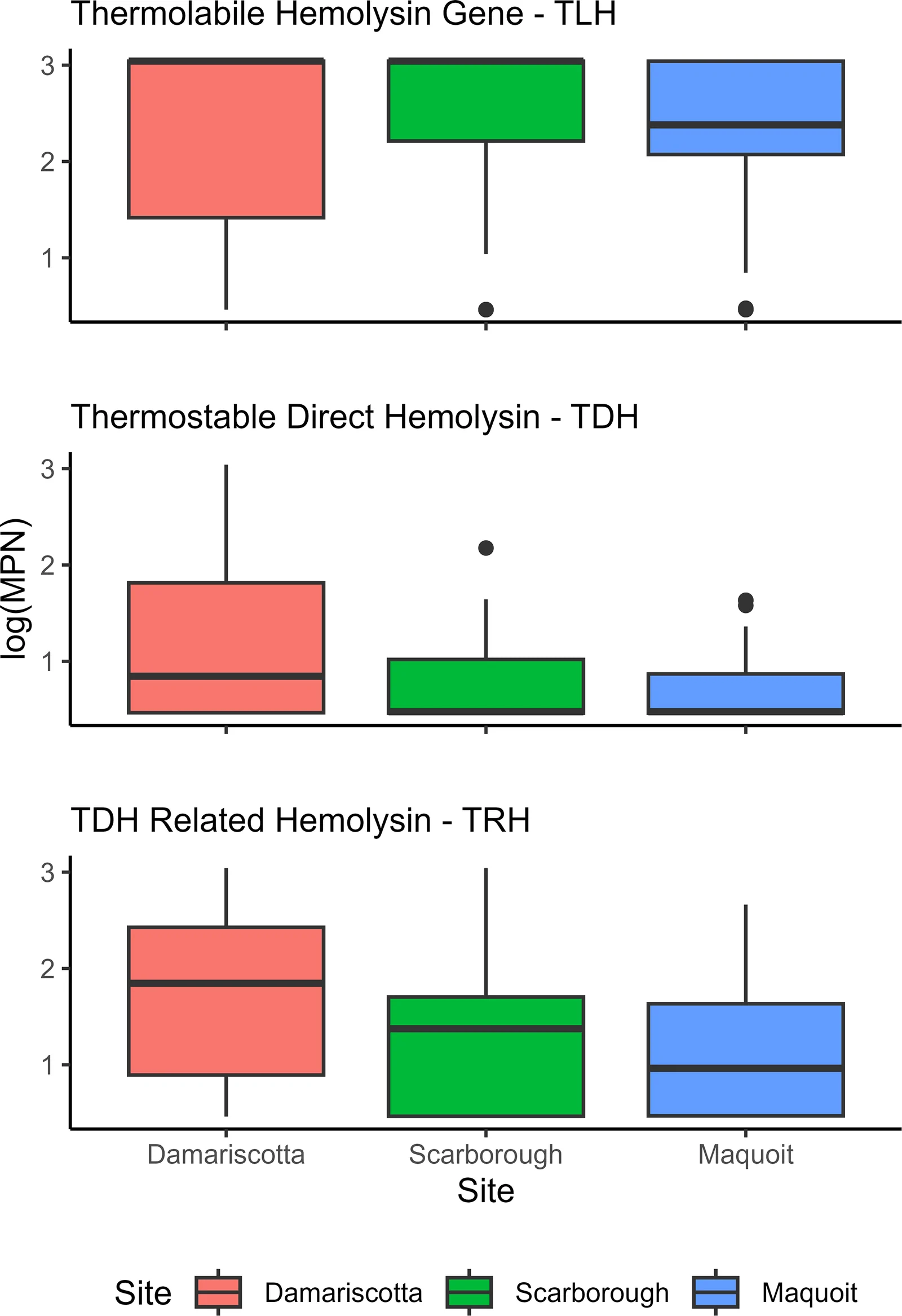
Box plot showing mean abundance of all three *Vibrio parahaemolyticus* genes in oysters from Scarborough River estuary, Maquoit Bay, and Damariscotta River estuary in Maine. The median value per site is represented as the solid black line.

Total *Vp*, represented by the *tlh* gene, did not provide peak abundance and accurate comparisons between sites as it was observed above the detection limit in most samples in the SRE (60%) and the DRE (53%). MB showed more seasonal change of *tlh*, exceeding the limit in only 15% of samples—although the lack of a sample in November limited the observations of when *tlh* decreased due to environmental conditions. Due to seasonal fluctuations of *tdh* and *trh* genes, we can more accurately compare the three sites and better focus modeling efforts on potential pathogenic strains. Potentially pathogenic *Vp* in the DRE was significantly more abundant than the other two estuarine systems. According to the nonparametric Kruskal-Wallis (K-W) rank-sum test, *tdh* abundance varied significantly by site even after accounting for environmental effects (*P* < 0.05). Between each site, *tdh* abundance in DRE and MB were significantly different from each other (*P* < 0.05), showing *tdh* abundance in DRE was 2.4 log(MPN g^−1^) higher than MB. *trh* by site was also significantly different (*P* < 0.05); specifically, DRE was significantly different from both MB (*P* < 0.05) and SRE (*P* < 0.05). Mean *trh* abundance in DRE was 2.4 log(MPN g^−1^) greater than MB and 1.8 log(MPN g^−1^) greater than SRE. *tdh* abundance in DRE had a median range of 0.845 log(MPN g^−1^) compared to 0.47 log(MPN g^−1^) in SRE and MB. *trh* abundance in DRE had a median range of 1.85 log(MPN g^−1^) compared to 1.37 log(MPN g^−1^) in SRE and 0.964 log(MPN g^−1^) in MB. *tlh* abundance in DRE and SRE had a median range of 3.0 log(MPN g^−1^) compared to 2.4 log(MPN g^−1^) in MB (Fig. 4).

GAMs were used to estimate gene abundance by constructing smooth functions of salinity (psu) and SST (°C), as well as a linear intercept to model site-level differences (Table 2). To test if accounting for variability in environmental relationships between sites improved explanatory power, we produced models with and without site-specific smooth functions. For *tdh*, we selected the model with the highest deviance explained and lowest Akaike information criterion (AIC), which included temperature site-specific smooth functions. For *trh*, we selected the model with the lowest AIC value, which did not include site-specific environmental information.

**TABLE 2:** Generalized additive model comparisons used to estimate potentially pathogenic *Vibrio parahaemolyticus* abundance using temperature, salinity, and site as covariates

Formula	Family	Link function	Smooth terms (*P* value)	*r*^2^ adjusted	REML^*a*^	Deviance explained (%)	AIC^*b*^
log(tdh_cq) ~ s(psu, *k* = 5) + s(temp, *k* = 5, by = site) + site	Tweedie	Identity	sal: 0.40	0.53	14.5	75.0	26.5
			temp Dam: <0.05				
			temp Maq: 0.07				
			temp Scarb: 0.07				
log(trh_cq) ~ s(psu, *k* = 5) + s(temp_u6, *k* = 5) + site	Gamma	Log	sal: 0.18	0.66	20.5	70.4	48.1
			temp_u6: <0.05				

^*a*^ REML, restricted maximum likelihood score.

^*b*^ AIC, Akaike information criterion.

**Fig 5:**
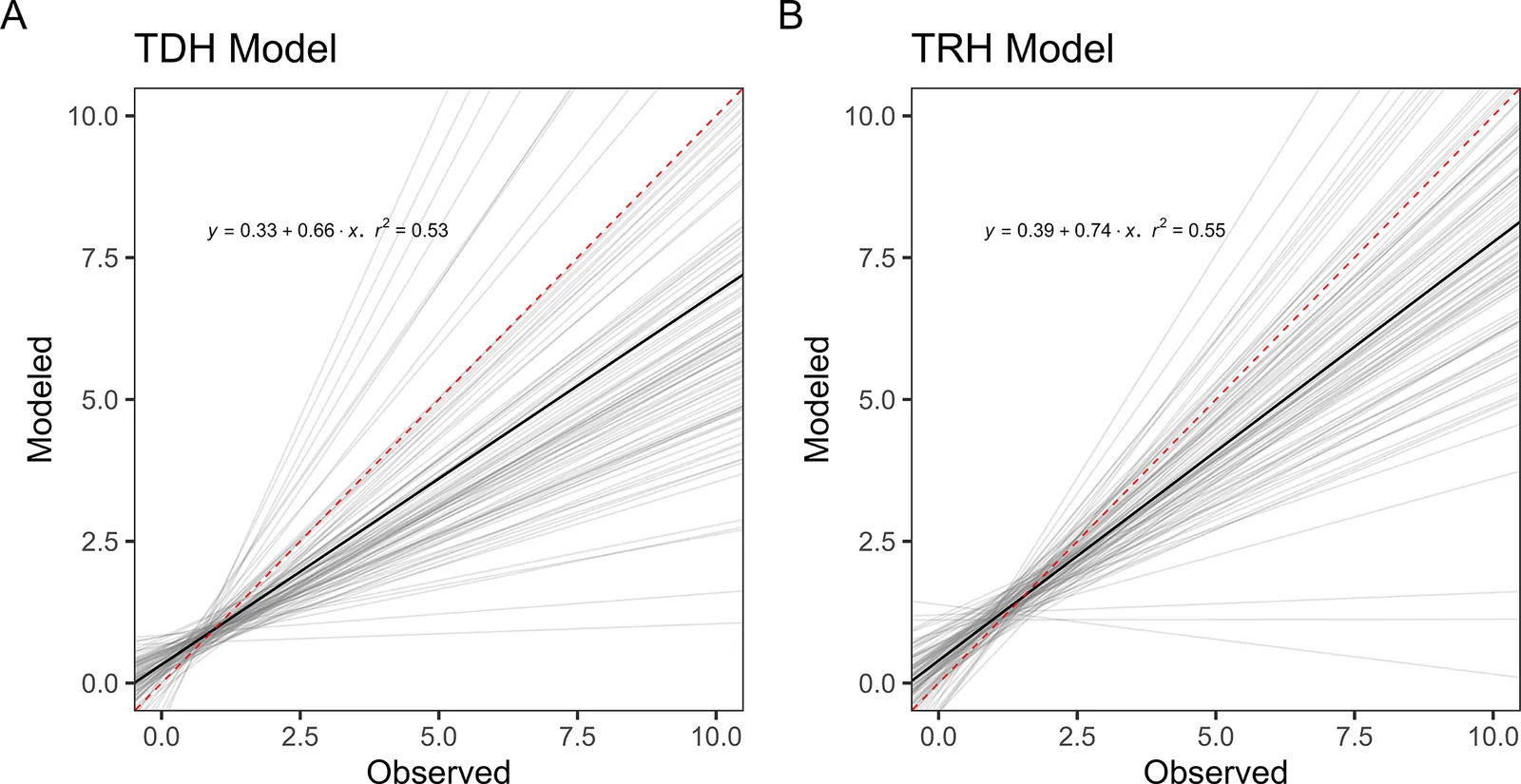
Model cross-validations for pathogenic *tdh* and *trh* genes log(MPN). (**A**) gam(log(*tdh*_cq) ~s(psu, *k* = 5) + s(temp, *k* = 5, by = site) + site). (**B**) gam(log(*trh_*cq) ~s(psu, *k* = 5) + s(temp_u6, *k* = 5) + site). Each model is cross-validated by comparing observed and predicted values of test data via linear regression 100 times. Mean intercept, slope, and *r*^2^ value of the regressions are presented on the panels. Individual validation fits are plotted in light gray. The mean slope-intercept line between observed and fitted values is plotted in black. The 1:1 line is plotted in dotted red.

To evaluate the predictive power of the final suite of models, we performed cross-validation comparing the observed and modeled gene abundance, simulating 100 linear regressions (Fig. 5). While a perfect model would show an intercept of 0, a slope of 1, and an *r*^2^ of 1, cross-validations depict a wide predictive range for both pathogenic genes depending on the training data set. Each model overpredicts slightly at low values, indicated by the positive intercept for an observed value of 0. Both models underpredict *Vp* abundance at higher abundances, with the *tdh* model underpredicting more than the *trh* model. When considering mean performance over the entire set of validations, the *tdh* model had an *r*^2^ of 0.53 compared to the 0.55 level for *trh*. The *trh* model had a slightly increased *r*^2^ compared to *tdh* due to more positive samples. Overall, the *trh* model is of higher predictive quality. These models overpredict at low abundances and underpredict higher abundances, bringing to light the limitations of these models for accurate forecasting (Fig. 5).

**Fig 6:**
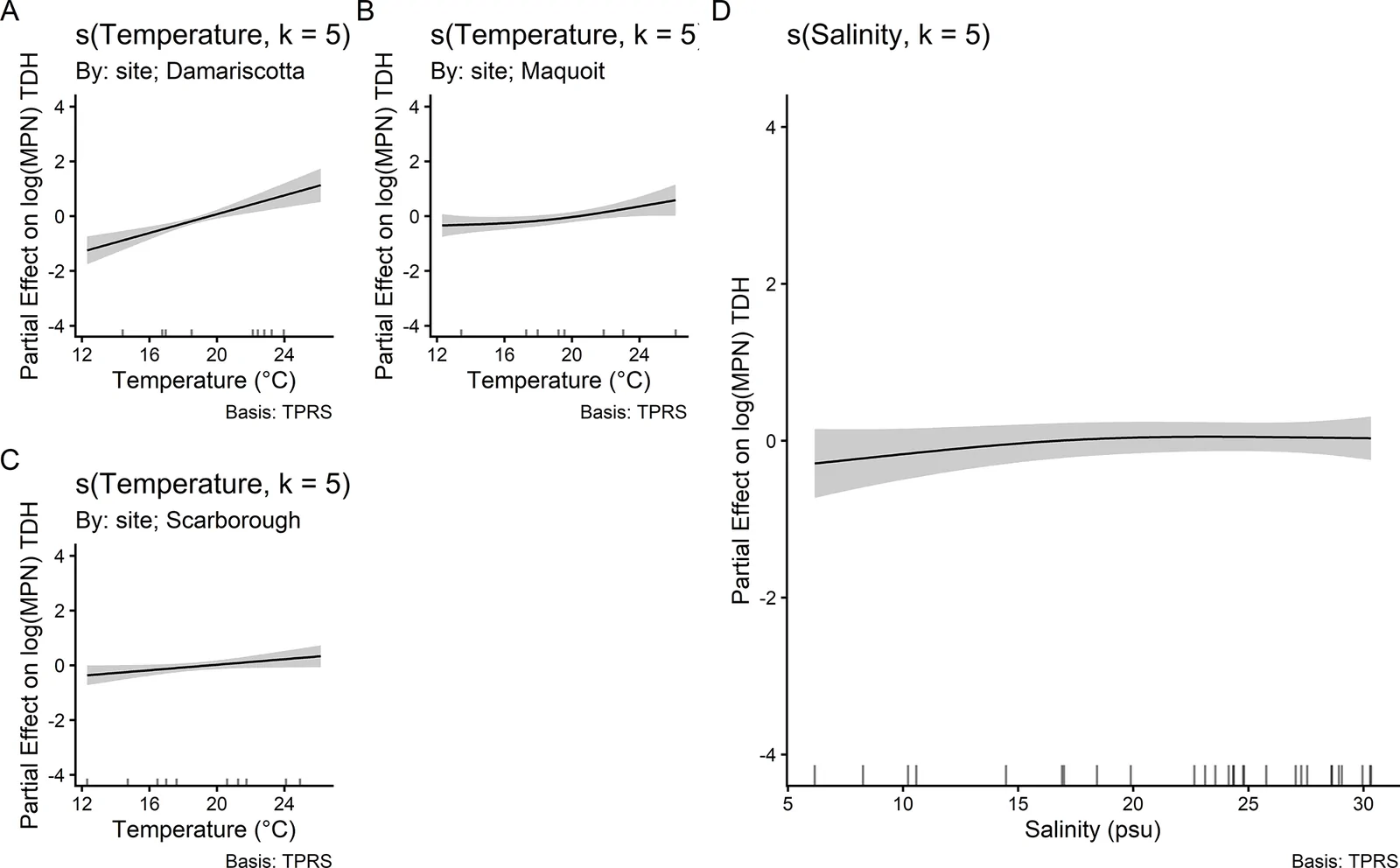
GAM partial effects on log(*tdh* MPN) for (**A**) the partial effect of temperature on *tdh* in Damariscotta (°C) and (**B**) the partial effect of temperature on *tdh* in Maquoit (°C). (**C**) The partial effect of temperature on *tdh* in Scarborough (°C). (**D**) The partial effect of salinity on *tdh* values. Smooth functions are fit using a thin plate regression spline (TPRS). The black line represents smoothed response relationships from GAMs, and the shaded gray area describes the 95% confidence interval estimates over the smooth function. Data rugs along the *x*-axis represent the density of observations.

Significant response covariates for *tdh* included site-specific immediate temperature (SST at the time of the sample), salinity (psu), and site. When we examined temperature by site, the smooth functions strongly suggested an underlying increasing relationship at each site. However, GAM results revealed a significant increase in *tdh* abundance in the DRE (*P* < 0.05; Table 2) as water temperature increases (Fig. 6), which indicated that the DRE is more responsive to temperature than the other two sites, with a roughly 4-log increase from 12°C to 26°C (Fig. 6). *tdh* abundance in MB and SRE was not significantly affected by temperature (*P* = 0.07; Table 2), although the smooth functions strongly suggest an underlying increasing relationship at these two sites. *tdh* abundance was still significant for temperature when site-specific information was not included (Table S1; *P* < 0.05), but it can be concluded that this positive relationship is driven by the DRE. Salinity was not a significant term for *tdh* in any model at any site. However, uncertainty in the relationship increases at lower values between 5 and 15 psu, suggesting there could be an underlying partial effect on *tdh* abundance and subsequent potentially pathogenic *Vp* abundance (Fig. 6). Despite the added complexity related to allowing the model to fit site-specific temperature relationships, the model deviance explained was highest and AIC was lowest in the *tdh* model that had this flexibility, indicating that temperature may have had different effects on *Vp* levels in each estuary. This is perhaps not surprising, given the potential that the temperature covariate was a proxy for processes affecting *Vp* abundance related to production, respiration, and grazing (Table 2).

**Fig 7:**
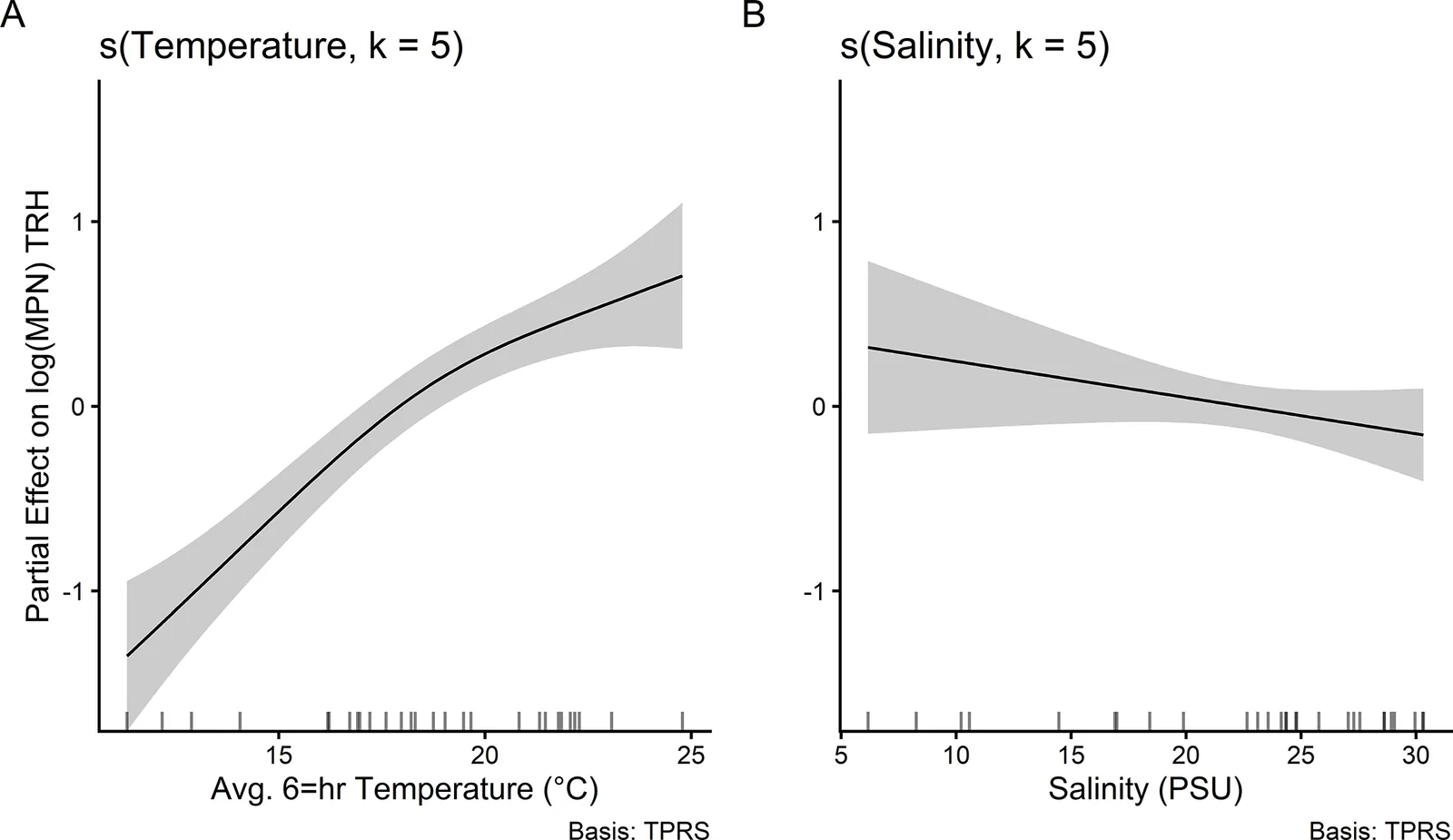
GAM partial effects on log(*trh* MPN) for (**A**) temperature (average 6 h prior to sampling) and (**B**) salinity. Smooth functions are fit using a thin plate regression spline (TPRS). The black line represents smoothed response relationships from GAMs, and the shaded gray area describes the 95% confidence interval estimates over the smooth function. Data rugs along the *x*-axis represent the density of observations.

Significant response covariates for *trh* included the average 6-hour SST prior to sampling, salinity (psu), and site. GAM results provide evidence of a significant increase in *trh* abundance (*P* < 0.05; Table 2) as temperature increased (Fig. 7), which indicated warming waters can promote potential pathogenic strains of *Vp*. When site-specific information was added, all three sites were also significant for temperature (Table S1; *P* < 0.05). When we examined salinity without site-specific information, it was not significant (*P* = 0.18), although the smooth functions strongly suggest an underlying negative relationship (Fig. 7). MB was the only site where *trh* was significantly affected by salinity (*P* < 0.05; Table S1). The model deviance explained is highest when these site-specific details are included, but cross-validations on either of these models resulted in unrealistic predictions, and instead, the model with the lowest AIC was selected (Fig. 6 and 7).

## DISCUSSION

*Vibrio*-related outbreaks associated with raw oysters have been occurring throughout the United States since the 1990s. Outbreaks have been historically more common in the Pacific Northwest, Texas, Alabama, New York, Connecticut, and Massachusetts (44, 62). In 2023, the state of Maine experienced its first *Vibrio*-related outbreak associated with Maine-grown oysters (63). Here, we describe the presence of potentially pathogenic strains of *Vp* throughout the warmer months, which can lead to potential illnesses if proper cold chain storage and harvest practices are not utilized. There was a strong seasonal trend in the abundance of total and potentially pathogenic *Vp* in Maine oysters throughout three of the most productive growing areas. *Vp* continues to become more common in the Northeast United States as the GoM warms.

*Vp* appears to be moving up the U.S. Atlantic coast and becoming more present in northern regions. In the mid-Atlantic, Audemard et al. (64) collected oysters in 2012 and 2014 and detected maximum *tdh* and *trh* in Chesapeake Bay oysters at 270 and 420 MPN g^−1^, with maximum water temperatures of 29°C. Here, we observed slightly lower maximum water temperatures but increased potentially pathogenic *Vp* in Maine 10 years later, using the same method. From 2009 to 2010, *tdh* and *trh* genes were detected in Rhode Island oysters, with higher abundance observed in coastal salt ponds compared to Narragansett Bay, with maximum detected abundance between 1,000 and 3,162 MPN g^−1^ when water temperature reached 24°C (65). Oysters from New Hampshire in 2009 were subjected to temperature abuse (28°C for 18–20 h) and tested for viable post-harvest processing strategies to reduce *Vibrio* species. *tdh*+/*trh*+ virulence genes were not detected in any oysters initially but were detected in temperature-abused oysters in August at 450 MPN g^−1^ (66). Urquhart et al. (34) collected oysters from 2008 to 2013 and tested for total *Vp* (*tlh*) in the Great Bay Estuary, NH, and determined that *tlh* ranged from 0.04 to 4,600 MPN g^−1^. The maximum *trh* abundance in this study was observed in the DRE and peaked when the temperature reached 23.9°C. Cox et al. (65) also noted a rapid increase in *Vp* once Rhode Island waters reached 19°C, which is also relatively consistent with our observations. Our results suggested that 16°C was the threshold temperature for the initial presence of *Vp* and maximum abundance when water temperature was above 20°C. As corroborated in previous studies (34, 35, 45, 55, 67), temperature is a major driver and significant covariate of *Vp* abundance in our models. These studies from the last two decades show the steady creep of potential pathogenic *Vp* up the coast and into northern New England; this study presents current seasonal abundance in the most northern sites sampled in Maine.

The long residence time of water in the DRE extends the season of high SST compared with adjacent estuaries in mid-coast Maine. While this characteristic is conducive to growing oysters, it also appears conducive to housing *Vp*. The DRE is the most productive oyster-growing area in northern New England. The upper third of the DRE is separated by natural bedrock, fostering unique characteristics that increase oyster growth, including higher residency times and warmer SST (~5°C) than the GoM overall (6, 68, 69). Even slight warming can affect the growth rates of *Vp*. For example, Harvell et al. (31) demonstrated that an average 1.5°C increase could significantly extend the seasonal period and range of *Vp*. In the DRE, we observed a warmer mean water temperature. This warmer seasonal temperature is likely the major driver behind increased *Vp* abundance compared to southern Maine sites.

In 2016, DRE oyster farmers approached the Maine Department of Marine Resources (ME DMR) and requested that a *Vp* control plan be developed and implemented, making it the first control area in Maine (70). Farmers in this area have understood the importance of minimizing *Vp* illness through regulations for almost 10 years; they know that single-source cases could close the DRE, negatively impacting the industry. Oyster harvest from the DRE is an order of magnitude higher than the other two systems; because bivalves are considered vehicles of potential pathogens (71) and may provide a reservoir of *Vp*, continuing to build our understanding of ecosystem dynamics in the most productive growing area in Maine is key. In light of increased illnesses, a more comprehensive study on baseline *Vp* data throughout the DRE is needed. After the recent doubling of Maine vibriosis cases during the time of this study and the first *Vibrio*-related outbreak involving oyster consumption, the *Vibrio* control plan for the state of Maine was expanded statewide starting in 2024 (63). At the time of this study, MB was not included in the *Vp* control plan and therefore not subject to *Vp* control regulations. Potentially pathogenic *Vp* was detected at all three sites, meaning oysters from any of these areas, if subjected to temperature abuse, could have resulted in an illness. Importantly, pathogenic genes were seen above the FDA reopening limit of 10 MPN g^−1^ during the warmest parts of the summer when oyster harvest is at its peak; during a future outbreak, it would not be surprising to find environmental abundance exceeding this limit. We now have comparative data to help inform management when future vibriosis illnesses or outbreaks occur. Instead of using the default reopening criteria of 10 MPN g^−1^ for both *trh* and *tdh*, as prescribed in the National Shellfish Sanitation Program Model Ordinance, in the future, we can consult previous environmental studies in order to reopen a growing area (58). With increasing water temperatures in the GoM, it is in the best interest of the industry to apply *Vibrio* regulations to all oysters harvested in Maine. Without post-harvest temperature control, bacteria can quickly proliferate in oysters; icing is a tested solution for limiting *Vp* growth (51).

Warming waters are one of the most obvious effects of climate change in the GoM, but other factors are also influencing *Vp* abundance. Increasing storms due to climate change may be of concern as changes in salinity may influence *Vp* dynamics. Out of the two potentially pathogenic markers used for regulatory guidance (i.e., *trh* and *tdh*), trends in *trh* abundance described in this study suggest that this gene was more responsive to salinity than both *tlh* and *tdh*, although low efficiency of the *tdh* probe could have resulted in underestimation and affected the comparison between these two genetic markers. As mentioned previously, trends in *tlh* (total *Vp*) could not be reasonably predicted due to frequent results that exceeded the maximum detection limit. On 19 September 2023, ME DMR issued flood closures along the coast of Maine in response to a rain event that resulted in decreased salinities across the state (Fig. 3) (61). *Vp* occupies a range of salinities (10–34 psu), and salinity levels in MB and SRE were reduced during this event due to river discharge; in both sites, we observed a secondary increase in *trh* abundance after it had started to decrease for the season (Fig. 2) (23, 24). Response curves for *tdh* show wide confidence intervals at low salinity. With the collection of more samples representing low-salinity conditions, we could gain a better understanding of this relationship. *tdh* and salinity were not significant but began to show a positive relationship below 20 psu; other studies, such as Johnson et al. (53), have shown *Vibrio* densities to increase with salinity up to a salinity optimum, detecting that optimum for *tlh* as 24 psu. In the coming years, vibriosis cases are expected to increase due to climate variabilities, such as heat waves, hurricanes, droughts, and floods (37). The continued collection of environmental variables along with *Vp* data is crucial in understanding how changes in the coastal environment may influence *Vp* abundance, which has predictive and regulatory implications.

Along with temperature and salinity monitoring, previous studies (20, 21, 35, 37, 55) have characterized the influence of pH, turbidity, chlorophyll, dissolved nitrogen, and dissolved oxygen on *Vp* abundance to explain trends and improve predictive models. *Vp* is driven by a combination of biological and physical parameters, but many of these parameters examined in previous studies show collinearity and are also driven mainly by seasonal temperature dynamics (35). Hartwick et al. (35) developed a robust modeling approach to forecast *Vp* abundance in oysters in Great Bay estuary in New Hampshire. Approximately half of *Vp* variability could be predicted using photoperiod in hours, sine and cosine of the day of study in harmonic regression, and the day of the study. Hartwick et al. (35) discuss the potential limitations with modeling relying only on environmental predictors due to ecological complexity and collinearity. With future monitoring, to more accurately model increased abundance of *Vp*, harmonic terms should be taken into consideration. Here, we focused on the influence of two primary variables, SST and salinity (21), on *Vp* gene expression. Our model results explain primary environmental drivers and suggest that SST is a key driver of *Vp* abundance; SST was the most significant term across all the models we tested. However, our model of *Vp* dynamics highlights that local site factors may also be playing a role. Even after using the model to control for temperature, *Vp* abundance in DRE was more sensitive to changes in temperature than the other two sites based on the calibrated smooth functions. Potential future directions include implementing a more mechanistic modeling approach and testing the influence of additional harmonic and environmental parameters. These model results cannot be used to accurately forecast *Vp* abundance, but they can provide evidence for the impact of the two most common environmental drivers of *Vp* in Maine waters. Improved modeling and forecasting capabilities specific to these unique systems will be beneficial to the public and the industry to help inform harvesters on conditions that may increase public health risks.

The ability to detect multiple *Vp* genes was crucial to the novelty of this study. Previous studies have looked only at total *Vp* using only the *tlh* gene (34, 35, 72, 73). Due to the maximum detection limit, had we only considered *tlh*, the only information gleaned would have been the effect of temperature on the seasonality of *Vp* abundance in 2023. Because *tdh* and *trh* facilitated observations of seasonal fluctuations in gene levels, they provided additional insight into the presence of potentially pathogenic strains. At times, we observed a large range between each triplicate sample for both *tdh* and *trh* (i.e., three orders of magnitude). Quality assurance steps did affect some of these triplicate samples, but it may be due to differences in bacterial accumulation influenced by individual oyster health, as described in previous studies (74, 75). As in previous studies conducted south of Maine, in this study, we observed increased *trh* abundance compared to *tdh* in oysters (37, 55, 65). The Minimum Information for Publication of Quantitative Real-Time PCR Experiments (MIQE) guidelines emphasize the necessity of calculating and reporting PCR efficiency in gene expression data (76). A low PCR efficiency was observed for the *tdh* probe throughout all 10 PCR runs, which may have impacted the detection of this target. Even with low efficiency, we still detected these potential virulence genes in environmental oyster samples, whereas earlier studies typically did not detect *tdh* and *trh* in environmental samples, and instead, these were mainly found in clinical strains that caused disease (77, 78). The increasing trend of environmental strains containing both hemolysin genes is thought to be due to increasing seawater temperatures; it has been determined that increased temperatures stimulate upregulation of virulence genes—meaning these increasingly favorable conditions are increasing the likelihood of *Vp* pathogenicity (71, 78–80).

*tdh* and *trh* are currently the most common predictive indicators of potential virulence (81). Even so, it is known that there are strains inducing acute gastroenteritis without the presence of *tdh* and *trh*. For example, Yu et al. (82) reported severe foodborne infections lacking these genes. Ottaviani et al. (83) isolated cytotoxic and adhesive strains that lacked these common virulent genes. Clinical strains that lack *tdh* and/or *trh* stress the need for additional testing when monitoring for pathogenicity. Studies discuss the lack of understanding and additional factors behind *Vp* virulence (24). Virulence factors include Type III secretion systems, the ToxR locus and adhesion factors causing cytotoxicity and disease (81), and the ability of non-pathogenic environmental strains to acquire potential virulent genes through horizontal transfer via mobile genetic elements (71). The FDA method approved in 2015 is what state regulatory agencies use as “best available science”; it assumes that *tdh* and *trh* will confirm the presence of pathogenic strains. As more information becomes available, it would be beneficial if the FDA added criteria to their method. In the meantime, regulatory agencies should confirm pathogenicity with additional methods to accurately evaluate their growing areas. In the future, samples positive for potentially pathogenic markers should undergo whole-genome sequencing or be serotyped to determine similarity to confirmed outbreak strains.

The success of the shellfish aquaculture industry relies on the safety of the consumer. Here, we document the *Vp* abundance in oysters across some of the largest growing areas in northern New England and demonstrate the potential risk of insufficient control plans due to *Vp* abundance exceeding maximum detection limits when using an FDA method for baseline collection. With a booming oyster industry in Maine, showcased by more raw bars and aquaculture lease applications, coupled with increasing water temperatures, there will continue to be vibriosis cases. This baseline characterization of *Vp* abundance across southern and mid-coast Maine highlights the importance of the following recommendations for future monitoring efforts in the region: (i) more dilutions are necessary to detect maximum *Vp* abundance in the summer months, (ii) site variability not explainable by temperature conditions alone indicates that more growing areas should be sampled, (iii) additional environmental covariates should be collected such as coliforms, pH, dissolved oxygen, turbidity, and chlorophyll, and (iv) additional characterization should be done (e.g., genotyping/serotyping) to confirm strains and their pathogenicity.

### Conclusion

Understanding the factors influencing increased abundance of the human pathogen *Vp* in Eastern oysters is critical for developing successful risk management plans for the shellfish industry. This study addresses a lack of data on *Vp* in Maine oysters and is already informing the development of future oyster sampling and *Vibrio* monitoring programs in Maine. We observed differences between three productive aquaculture sites; the unique salinity ranges and seasonal temperature fluctuations resulted in site-specific baseline data of the seasonal abundance of both total and potentially pathogenic *Vp*. This study occurred during a summer of unprecedented vibriosis illnesses, which included the first outbreak and growing area closure associated with *Vibrio*, resulting in the implementation of a statewide *Vp* control plan during the summer of 2024. We observed potentially pathogenic *Vp* in all three sites, one of which was not under *Vp* control during the summer of 2023. This study gives the region’s initial baseline data to use in the case of future illness outbreaks and growing area closures while focusing on the ecology of these bacteria and what factors are contributing to their distribution in Maine. Although *Vp* abundances vary seasonally between growing areas, the warming waters in the GoM are a clear contributor to increasing *Vibrio*-related illnesses. These data inform oyster harvesters and policy makers while preparing for a future in which the GoM continues to change and *Vibrio*-related illnesses persist.

## MATERIALS AND METHODS

### Study sites and sample collection

**Fig 8:**
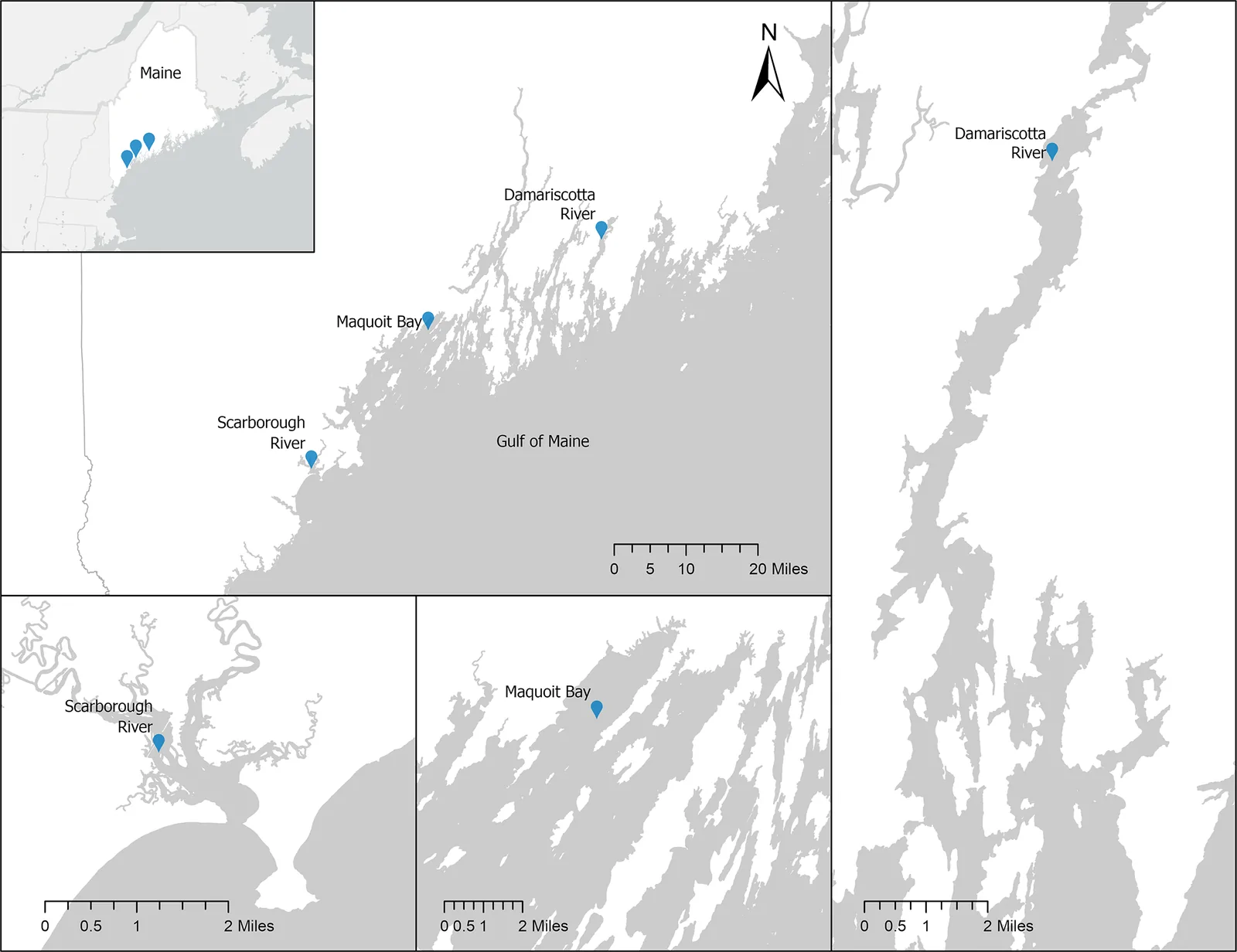
Locations of sample sites in southern and mid-coast Maine. Esri, NASA, NGA, USGS. Sources: Esri, TomTom, Garmin, FAO, NOAA, USGS, ©OpenStreetMap contributors, and the GIS User Community.

We targeted three representative growing areas in the GoM: the SRE, MB, and the DRE (Fig. 8). SRE, a *Vibrio* control area, is fringed by the largest salt marsh in the state; MB, a non-*Vibrio* control area, is representative of arcuate embayments in southern to mid-coast Maine; and the DRE, the first *Vibrio* control area, the largest oyster-growing area in northern New England (6), represents a long narrow drowned river valley common in mid-coast Maine. These three sites are distinctly different coastal ecosystems that are common shellfish-harvesting areas; both SRE and DRE have been linked to vibriosis illnesses, while MB has not, acting as a potential control site. Eastern oysters (*Crassostrea virginica*) were collected from May to November 2023. Each site was sampled monthly—increasing to biweekly in the warmer months (July–September). Oysters were harvested within 1 h of low tide for consistency, except the sample on 22 August 2023 from the DRE, which was collected 5 h after low tide. A total of 10–12 oysters were collected in triplicate from floating oyster cages (MB and DRE) and floating bags (SRE), and samples were immediately placed in labeled bags in a cooler with ice packs and a thermometer. Samples were transported to the ME DMR Lab in Boothbay Harbor within 4 h of harvest, arriving below 10°C. Continuous water temperature and conductivity readings were collected every hour with Hobo U24 data loggers attached to floating oyster cages. During each sample collection, a calibrated YSI meter was used to measure immediate water temperature and salinity and to calibrate the Hobo U24 data logger at the end of the field season.

### *Vp* enrichment

Samples were prepared for the three-tube MPN enrichment method (84). Specifically, the outer shells of oysters were scrubbed with a sterilized brush under running potable water and set on clean paper towels. Triplicate samples of 10–12 oysters were aseptically shucked (~200 g) and homogenized for 90 s in a blender with a 1:1 dilution using phosphate-buffered saline (PBS). Initial dilutions were made in PBS, and from those dilutions, 1:10, 1:100, and 1:1,000 dilutions were prepared in triplicate in alkaline peptone water (APW). Negative process control of APW was added to the tray along with a *Vp* positive 1:1,000 dilution acquired from culture (ST36 serotype 04:K12 strain MAVP-26) (85). All tubes were incubated for 18–24 h at 35°C. Following incubation, aliquots from tubes with visible growth were boiled for 10 min at 100°C, then immediately plunged into an ice slurry. Samples were centrifuged for 2 min at 1,500 rpm. Supernatant was stored at –20°C until tested by real-time PCR (14, 84).

### Sample analysis

Samples were analyzed using presence/absence qPCR to determine total *Vp* (*tlh* gene) and potentially pathogenic *Vp* (*tdh* and *trh* genes) by multiplex qPCR using the ABI 7500 Fast PCR system (Life Technologies, Foster City, CA, USA) and 96-well plates (ThermoFisher). Each 25 μL reaction mixture contained a final concentration of 1× PCR buffer (Invitrogen, Carlsbad, CA, USA), 5 mM MgCl_2_ (Invitrogen), 1.5 μL Platinum *Taq* polymerase (Invitrogen), 300 nM deoxynucleoside triphosphate mix (equal concentrations) (Thermo Scientific), 0.06 μL ROX reference dye (Invitrogen), 2 μL internal amplification control DNA (BioGX, Birmingham, AL, USA), 200 nM *tlh* primer 884F, 200 nM *tlh* primer 1091R, 100 nM primer *tdh* 89F, 100 nM primer *tdh* 321R, 300 nM primer *trh* 20F, 300 nM primer *trh* 292R, 75 nM *tdh* probe FAM 268-20, 75 nM *trh* probe 133-23, 75 nM IAC primer 46F, 75 nM IAC primer 186R, 150 nM *tlh* probe 1043C JOE, 150 nM IAC probe 109 Cy5 (Table 3), and 2 μL of boiled DNA template as previously described by Jones et al. (14). Two replicates of a negative template control were made by adding 2 μL of PCR-grade water to their designated well, and two positive controls were included on the plate by adding 2 μL of a positive sample (ST36 serotype 04:K12 strain MAVP-26) (85) to the sample wells. Plates were sealed with adhesive film (ThermoFisher) and centrifuged to ensure all reagents were at the bottom of the well. Cycling conditions were 95°C for 60 s, followed by 45 cycles of 95°C for 5 s, 59°C for 45 s, and a “read” stage of 59°C for 45 s. Default instrument settings were used with the threshold set at 0.02 and the background end cycle set at 10. All primers and probes were purchased from Integrated DNA Technologies (Coralville, IA, USA) or Applied Biosystems, originally described by Nordstrom et al. (86).

**TABLE 3:** Oligonucleotide sequences ordered from ThermoFisher and Integrated DNA Technologies (NSSP Guide for the Control of Molluscan Shellfish: 2019 Revision)

Primer/probe ID	Oligonucleotide sequence
tdh_269-20	6FAM-5′-TGACATCCTACATGACTGTG-3′-MGBNFQ
trh_133-23	NED/TET-5′-AGAAATACAACAATCAAAACTGA-3′-MGBNFQ
tlh_1043	JOE/TEXAS RED-5′-CGCTCGCGTTCACGAAACCGT-3′-BHQ2
IAC_109	CY5-5′-TCTCATGCGTCTCCCTGGTGAATGTG-3′-BHQ2
trh_20F	5′-TTGCTTTCAGTTTGCTATTGGCT-3′
trh_292R	5′-TGTTTACCGTCATATAGGCGCTT-3′
tdh_89F	5′-TCCCTTTTCCTGCCCCC-3′
tdh_321R	5′-CGCTGCCATTGTATAGTCTTTATC-3′
tlh_884F	5′-ACTCAACACAAGAAGAGATCGACAA-3′
tlh_1091R	5′-GATGAGCGGTTGATGTCCAAA-3′
IAC_46F	5′-GACATCGATATGGGTGCCG-3′
IAC_186R	5′-CGAGACGATGCAGCCATTC-3′

Quantitative PCR to detect the presence of nucleic acid template was used for pathogen diagnostics (87). Due to the generation of the Ct (cycle threshold) value, which represents the cycle in which the sample’s reaction curve intersected the set threshold, the sample was initially considered positive. The criteria for a valid PCR run included a Ct value for the positive control, no Ct value for the negative control, and Ct values for internal amplification control (IAC) in all samples. If the IAC and target were negative, the result was considered invalid and reprocessed. Raw exported qPCR data were analyzed through real-time PCR Data Markup Language (RDML) with LinRegPCR software to determine standardized PCR information (88, 89). In accordance with RDML and MIQE guidelines, Cq (quantification cycle) is the term used in this study as it is the standardized qPCR nomenclature (87, 88). Any Cq values >40 were not included due to implied low efficiency (87). LinRegPCR software was used to perform a baseline correction on raw fluorescence data, identify the linear portion of the amplification curve, and, using that slope, calculate efficiency based on a standard formula (89). Samples with PCR efficiencies less than 1.7 were still considered valid to maintain temporal coverage across study sites. Any sample that did not display a sigmoidal amplification curve or had no values in the log phase was considered invalid. qPCR Cq results were used with standard MPN tables to estimate total and potentially pathogenic *Vp* (90). Based on the range of dilutions analyzed and the number of tubes per dilution (*n* = 3 tubes), the detection limit given using the MPN tables was 3.0 MPN g^−1^.

### Statistical analysis

Hobo U24 data logger outputs were calibrated using the R package *DriftR*. MPN analyses were based on the triplicate mean of each sample. Due to the non-normal distribution of the data, a K-W rank-sum test with the Benjamini-Hochberg procedure was used to determine significant differences between group medians of *Vp* abundance within sample sites and environmental characteristics (91, 92). Mean MPN values for *Vp* abundance were log-transformed prior to statistical analyses to approximate normality. Samples below the detection limit of 3 MPN g^−1^ were assigned a value of 2.9 MPN g^−1^, and samples above the detection limit of 1,100 MPN g^−1^ were assigned a value of 1,101 MPN g^−1^ for analyses. GAMs were used to examine the influence of environmental covariates on *tlh*, *trh,* and *tdh* gene concentrations. The R “gam” function was used for analysis (93). Previous 6, 12, 24, 48, 72, 96, and 144 h temperature and salinity values were evaluated to confirm if there was an effect on *Vp* abundance. The model was developed with all potential environmental covariates and then refined by removing insignificant terms. Each model was evaluated based on deviance explained and AIC. *tdh* and *trh* models were cross-validated using identical methods. For each model, cross-validation was performed using a random split of 66% of the data for training and 34% for testing. A linear model was created for each test validation to show how well-modeled values matched observed values. The process was iterated 100 times (94, 95). Only samples with corresponding environmental data were used for modeling. All statistical analyses were performed in R 4.4.1 (96). Significance for all analyses was determined by a *α* < 0.05 (93).

## Data Availability

The data used in the findings of this paper are publicly available on Zenodo at https://doi.org/10.5281/zenodo.17436903 (97).

## SUPPLEMENTAL MATERIAL

Table S1 aem.00168-26-s0001.pdf Additional model comparisons.

ASM does not own the copyrights to Supplemental Material that may be linked to, or accessed through, an article. The authors have granted ASM a non-exclusive, world-wide license to publish the Supplemental Material files. Please contact the corresponding author directly for reuse.
