# Supplementary material for: Seasonality of Vibrio parahaemolyticus in the Eastern oyster, Crassostrea virginica, along the coast of Maine, USA
Source: Appl Environ Microbiol. 2026 Jul 31;92(8):e00168-26. doi: 10.1128/aem.00168-26 (PMC13488352; doi:10.1128/aem.00168-26)
Supplement: Supplementary file 1 [file aem.00168-26-s0001.pdf]

Supplemental figures.

Table 1.Generalized Additive Model comparisons used to estimate potentially pathogenic *Vibrio parahaemolyticus* abundance using temperature, salinity, and site as covariates.

Formula	Family	Link Function	Smooth terms (p value)	R sq adjusted	REML	Dev Explained (%)	AIC
log(tdh_cq) ~ s(psu, k = 5) + s(temp, k = 5) + site	Tweedie	Identity	sal: 0.43	0.45	13.6	67.90%	27.3
			temp: < 0.05				
log(tdh_cq) ~ s(psu, k = 5) + s(temp, k = 5, by = site) + site	Tweedie	Identity	sal: 0.40	0.53	14.5	75.00%	26.5
			temp Dam: < 0.05				
			temp Maq: 0.07				
			temp Scarb: 0.07				
log(tdh_cq) ~ s(psu, k = 5, by = site) + s(temp, k = 5) + site	Tweedie	Identity	temp: <0.05	0.42	13.9	67.40%	32.7
			sal Dam: 0.92				
			sal Maq: 0.63				
			sal Scarb: 0.51				
log(trh_cq) ~ s(psu, k = 5) + s(temp_u6, k = 5) + site	Gamma	Log	sal: 0.18	0.66	20.5	70.40%	48.1
			temp_u6: < 0.05				
log(trh_cq) ~ s(psu, k = 5) + s(temp_u6, k = 5, by = site) + site	Gamma	Log	sal: 0.14	0.63	22	71.40%	52
			temp Dam: < 0.05				
			temp Maq: < 0.05				
			temp Scarb: < 0.05				
log(trh_cq) ~ s(psu, k = 5, by = site) + s(temp_u6, k = 5) + site	Gamma	Log	temp u_6: < 0.05	0.64	19.4	74.30%	48.2
			sal Dam: 0.55				
			sal Maq: < 0.05				
			sal Scarb: 0.95				
